# Supplementary material for: Complexome profiling of the Chlamydomonas psb28 mutant reveals TEF5 as an early PSII assembly factor
Source: Plant Cell. 2025 Mar 17;37(6):koaf055. doi: 10.1093/plcell/koaf055 (PMC12164590; doi:10.1093/plcell/koaf055)
Supplement: koaf055_Supplementary_Data [file koaf055_supplementary_data.zip › psb28_interactive.zip!/plots/intensity/qualityPlots/peptides/PSBP1_Cre12.g550850.t1.2.html]

### Cre12.g550850.t1.2

Trivial name: PSBP1  
SeqCoverage [median]: 68.20  
Mass: 25.90 kDa  
  
 

| Sequence | MissedCleavage | Mass [Da] | wtIntensity | muIntensity | Score |
| --- | --- | --- | --- | --- | --- |
| AIADFGSQDK | 0 | 1050.50 | 647382.11 | 211107.62 | 213.85 |
| AIADFGSQDKFLESVSYLLGK | 1 | 2287.17 | 2644.93 | 0.00 | 107.33 |
| EAMGAFDSFTVV | 0 | 1272.57 | 884179.84 | 180140.99 | 205.70 |
| ENDFPGVILR | 0 | 1158.60 | 131949.55 | 162557.49 | 257.27 |
| FLESVSYLLGK | 0 | 1254.69 | 189819.39 | 182406.61 | 243.29 |
| GKTYYK | 1 | 758.40 | 11426.45 | 1108.25 | 148.21 |
| GKTYYKYELLVR | 2 | 1531.84 | 90448.18 | 30411.74 | 158.59 |
| HQLIGATVGSDNK | 0 | 1338.69 | 69314.86 | 56149.47 | 172.21 |
| HQLIGATVGSDNKLYIIK | 1 | 1969.10 | 39120.12 | 27034.86 | 212.92 |
| IQIGDKR | 1 | 828.48 | 466795.95 | 121715.14 | 180.00 |
| KAIADFGSQDK | 1 | 1178.59 | 1502.56 | 0.00 | 107.06 |
| KEAMGAFDSFTVV | 1 | 1400.66 | 416276.83 | 96191.40 | 128.01 |
| QAYSGETQSEGGFAPNR | 0 | 1797.79 | 717109.27 | 200326.86 | 427.40 |
| QAYSGETQSEGGFAPNRVSAASLLDVSTTTDKK | 2 | 3414.65 | 2475.43 | 0.00 | 79.41 |
| SADGDEGGRHQLIGATVGSDNK | 1 | 2183.02 | 193797.40 | 83720.64 | 161.58 |
| SADGDEGGRHQLIGATVGSDNKLYIIK | 2 | 2813.43 | 241824.40 | 75090.30 | 192.75 |
| SGFVPYAGDGFALLLPAK | 0 | 1821.97 | 167306.06 | 83561.69 | 169.09 |
| TYYKYELLVR | 1 | 1346.72 | 390734.73 | 269888.27 | 189.58 |
| VSAASLLDVSTTTDK | 0 | 1506.78 | 323321.07 | 180472.31 | 331.54 |
| VSAASLLDVSTTTDKK | 1 | 1634.87 | 1608358.00 | 943679.14 | 222.45 |
| VSAASLLDVSTTTDKKGK | 2 | 1819.99 | 219534.49 | 59775.36 | 174.35 |
| WNPSKENDFPGVILR | 1 | 1770.91 | 78000.36 | 53953.83 | 144.12 |
| YEDNFDAVNNLVVIAQDTDK | 0 | 2282.07 | 1244.71 | 0.00 | 110.56 |
| YEDNFDAVNNLVVIAQDTDKK | 1 | 2410.17 | 136962.37 | 24738.31 | 260.24 |
| YELLVR | 0 | 791.45 | 648495.58 | 382515.39 | 156.93 |
|  |  |  | 7680024.6 | 3426545.7 |  |
